# Resurfacing Threats: Metastatic Ossifying Fibromyxoid Tumor Emerging After Almost Two Decades

**DOI:** 10.1002/gcc.70091

**Published:** 2025-11-12

**Authors:** Mario Ambros, Bernadette Liegl‐Atzwanger, Karl Kashofer, Andreas Leithner

**Affiliations:** ^1^ Department of Orthopaedics and Trauma Medical University of Graz Graz Austria; ^2^ Diagnostic and Research Institute of Pathology Medical University of Graz Graz Austria

**Keywords:** copy number variation, DNA methylation, EP400:PHF1, mesenchymal tumor, OFMT, ossifying fibromyxoid tumor

## Abstract

**Background:**

Ossifying fibromyxoid tumor (OFMT) is an extremely rare mesenchymal tumor of uncertain differentiation having a potential for local recurrences and metastasis. OFMT can be classified as typical, atypical, and malignant tumors based on nuclear grade, cellularity, and mitotic rate. However, predicting the biological behavior remains challenging. We report one of these challenging cases of OFMT with metastases after 19 years. The primary tumor did not show morphologic characteristics of malignancy. We performed targeted RNA sequencing, copy number variation (CNV) analysis on all lesions and additional DNA methylation profiling.

**Presentation of the Case:**

Herein we report the case of a 66‐year‐old Caucasian female patient diagnosed in 2004 with an OFMT on her back that was surgically removed. In August 2023, the patient presented with two new soft tissue lesions in the right lower thigh and left gluteal area. Biopsy has been performed on both lesions. Pathology reports demonstrated an OFMT with an *EP400:PHF1* fusion. Both tumors were confirmed to represent the identical entity. Surgical removal of both tumors was performed with clear margins. The presence of late sequential metastases 19 years after initial resection of an OFMT, without any signs of malignancy after pathology review of the 2004 lesion was suggested. In December 2023, a potential fourth similar mass was detected with MRI and a full body PET‐CT within the distal left semimembranosus muscle. A biopsy of this lesion has not been performed yet. It is currently being closely monitored through regular MRI examinations with contrast agents at intervals of 3 months.

**Conclusion:**

This case demonstrates the rare unpredictable potential of even typical OFMT to develop multiple soft tissue metastases after 19 years. Therefore, clinicians need to be aware of the metastatic potential of this tumor. Comprehensive and prolonged postoperative follow‐up is necessary to track local recurrences and metastases.

## 1 | Introduction

Ossifying fibromyxoid tumor (OFMT) is an exceedingly rare soft tissue tumor with uncertain differentiation [1]. Lesions often occur in the subcutaneous tissue but are also seen in the skeletal muscles of the extremities. Evidence suggests that OFMT has the unpredictable capacity for recurrence and metastasis. The occurrence of this is often postponed and might manifest itself even 20 years or more after the initial excision [2].

The tumor often manifests as a painless, gradually expanding, resilient, firm mass. From a radiological perspective, OFMT often appears as a well‐defined, lobulated mass, sometimes with partial calcification around its edges [3].

With a thick fibrous capsule or pseudocapsule, OFMTs are typically well‐defined under the microscope [2, 4, 5]. The tumor often shows a fibrous capsule/pseudocapsule and fibrous septa extending into the tumor, resulting in a multinodular appearance. In many cases, typically a peripheral shell of woven or lamellar bone, which may be complete or incomplete can be seen [2]. However, cases lacking the typical shell of bone are reported as non‐ossifying fibromyxoid tumor [6]. Fibrous septa may contain bone as well. Although the tumor may appear circumscribed when viewed at low magnification, it has the ability to penetrate the capsule and create nodules in the surrounding soft tissue [5]. The tumor consists of lobules made up of cells that are uniform in shape, ranging from round to spindle‐shaped. These cells have bland, round to ovoid nuclei surrounded by narrow pale, eosinophilic cytoplasm [2]. Tumor cells often form arrangements in the shape of cords, nests, or sheets, which are surrounded by stroma that may vary in its fibrous or myxoid composition. The level of cellularity ranges from low to moderate to high. A risk stratification for OFMTs has been proposed by Folpe et al. and is summarized in Table 1 [2].

**TABLE 1:** Risk stratification for OFMT according to Folpe et al. [2].

	Diagnostic criteria	Local recurrences	Metastases
Typical OFMT	Low nuclear grade *and* low cellularity *and* mitotic rate < 2/50 HPF	12%	4%
Atypical OFMT	Tumors deviating from typical OFMT but not meeting criteria for malignant OFMT	13%	6%
Malignant OFMT	High nuclear grade *or* high cellularity *and* mitotic activity of greater than 2/50 HPF	60%	60%

Abbreviation: HPF = high power field.

Typical OFMTs generally have a low level of mitotic activity of < 2/50 HPF [2, 7]. S100 is positive in about two thirds of OFMTs; however, diffuse immunoreactivity is uncommon. Desmin expression is seen in approximately 50% of cases [8]. They also exhibit low cellularity and low nuclear grade [2].

Malignant OFMTs are defined by high nuclear grade or high cellularity, along with more than 2 mitoses per 50/HPF that have a substantial risk of metastasis. Within clinically malignant lesions, the presence of bone or osteoid deposition within tumor cell nodules can be observed [2, 9].

OFMTs with histological characteristics that slightly deviate from the typical OFMT, but do not meet the criteria for the malignant subtype such as increased nuclear grade or high cellularity with up to > 2/50 HPF, can be classified as atypical OFMT. These OFMT do not meet the criteria for typical OFMT and also do not meet the criteria for “malignant” OFMT [2].

The primary sites of metastasis commonly include the lungs and soft tissues [1].

## 2 Case Presentation

**FIGURE 1:**
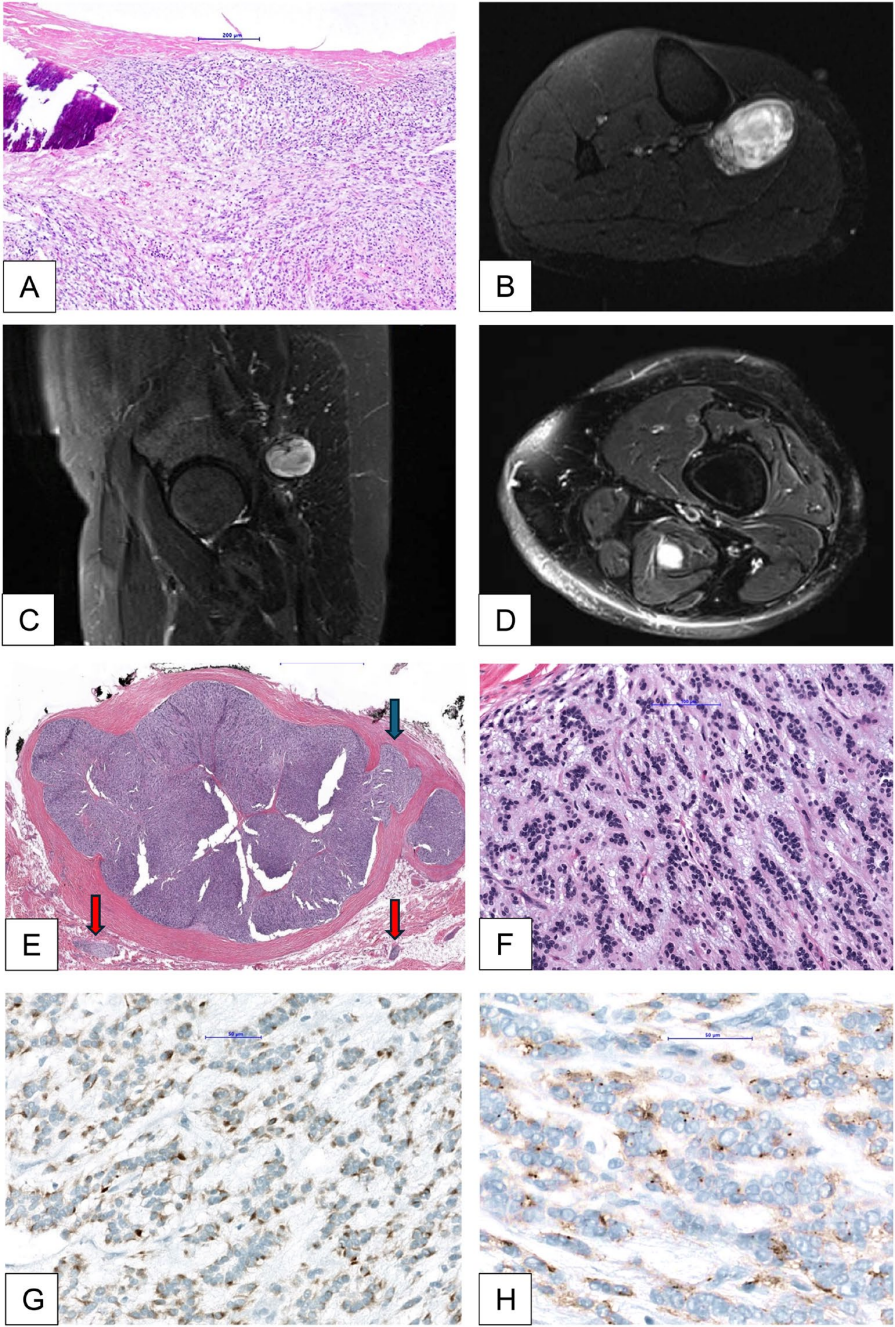
Magnetic resonance imaging of OFMTs and histologic workup of the tumor tissue. (A) OFMT left infrascapular region of 2004. Fibrous capsule, metaplastic bone at the periphery, monotonous tumor cells, lacking cytologic atypia and mitotic activity. (B) MRI of the right lower leg (tumor B). (C) MRI of the gluteal region in sagittal plane (tumor C). (D) MRI of the left upper thigh in axial (not yet operated on). (E) Hematoxylin and eosin stain with small tumor nodules in fat (tumor C) (HE) overview (tumor C). (F) Hematoxylin and eosin stain (HE) bland ovoid tumor cells in a collagenous/myxoid matrix (tumor C). (G) Desmin expression in tumor cells (tumor C). (H)Epithelial membrane antigen (EMA) expression in tumor cells (tumor C).

The patient, a Caucasian 66‐year‐old female, was first hospitalized in a peripheral hospital in February 2004 for resection of a soft tissue lesion in the left infrascapular region. A fibrolipoma was clinically suspected and subsequently excised. The pathology assessment demonstrated a 5 × 4.5 × 3 cm lesion subsequently diagnosed as an OFMT (Figure 1A). The OFMT was resected with close margins. A re‐resection was performed. Microscopically, no tumor cells were present in the re‐resected sample. Three chest x‐rays were performed over time (2004, 2010, and 05/2022), all of which showed no signs of any lung metastases.

The patient received treatment for a vertebral fracture in July 2022. During the course of treatment, a subcutaneous tumor was discovered in the left gluteal region (Figure 1C), and subsequently, another lesion was found in the proximal right lower leg (Figure 1B). Histopathological examination was conducted on both lesions using core needle biopsies (core biopsy cylinder).

The diagnosis of OFMT with an *EP400:PHF1* fusion in both lesions, a characteristic hallmark of OFMTs was confirmed.

In September 2023, a tumor, measuring 7 × 5 × 4.5 cm, was surgically removed from the right lower leg with clear margins. Histologically, the tumor was well circumscribed and surrounded by a thick fibrous capsule. The cells were embedded in a partly myxoid and partly collagenous matrix. The tumor cells had round, uniform nuclei and were surrounded by a narrow rim of pale eosinophilic cytoplasm. The tumor cells were arranged in strands and small nests. Immunohistochemically, the tumor showed focal positivity for the S‐100 protein and weak positivity for desmin. Criteria indicating malignancy have not been observed.

In November 2023, a second tumor located in the left gluteal region measuring 9.5 × 5.5 × 3.5 cm was surgically resected with R0 margins. The histology was similar to that of the OFMT in the right lower leg. Focally, bone formation was detected at the edge of the tumor, and single mitoses were present not reaching > 2/50 HPF. The tumor invaded the fibrous capsule (arrow blue on Figure 1E) and small satellite nodules in the adjacent soft tissue (arrow red on Figure 1E).

Control imaging identified an additional lesion in the semimembranosus muscle on the left side in December 2023 (Figure 1D), which appeared radiologically similar to the prior lesions. The patient was presented with the option of surgical intervention, but declined the procedure. Hence, we recommended continuing to closely monitor the situation using whole‐body contrast MRI scans every 3 months to exclude the presence of any more neoplasms, as previously mentioned. A total body 18F‐FDG PET/CT was performed in January 2024. No pneumonic consolidations and no suspicious intrapulmonary nodules were detected in the chest CT.

**FIGURE 2:**
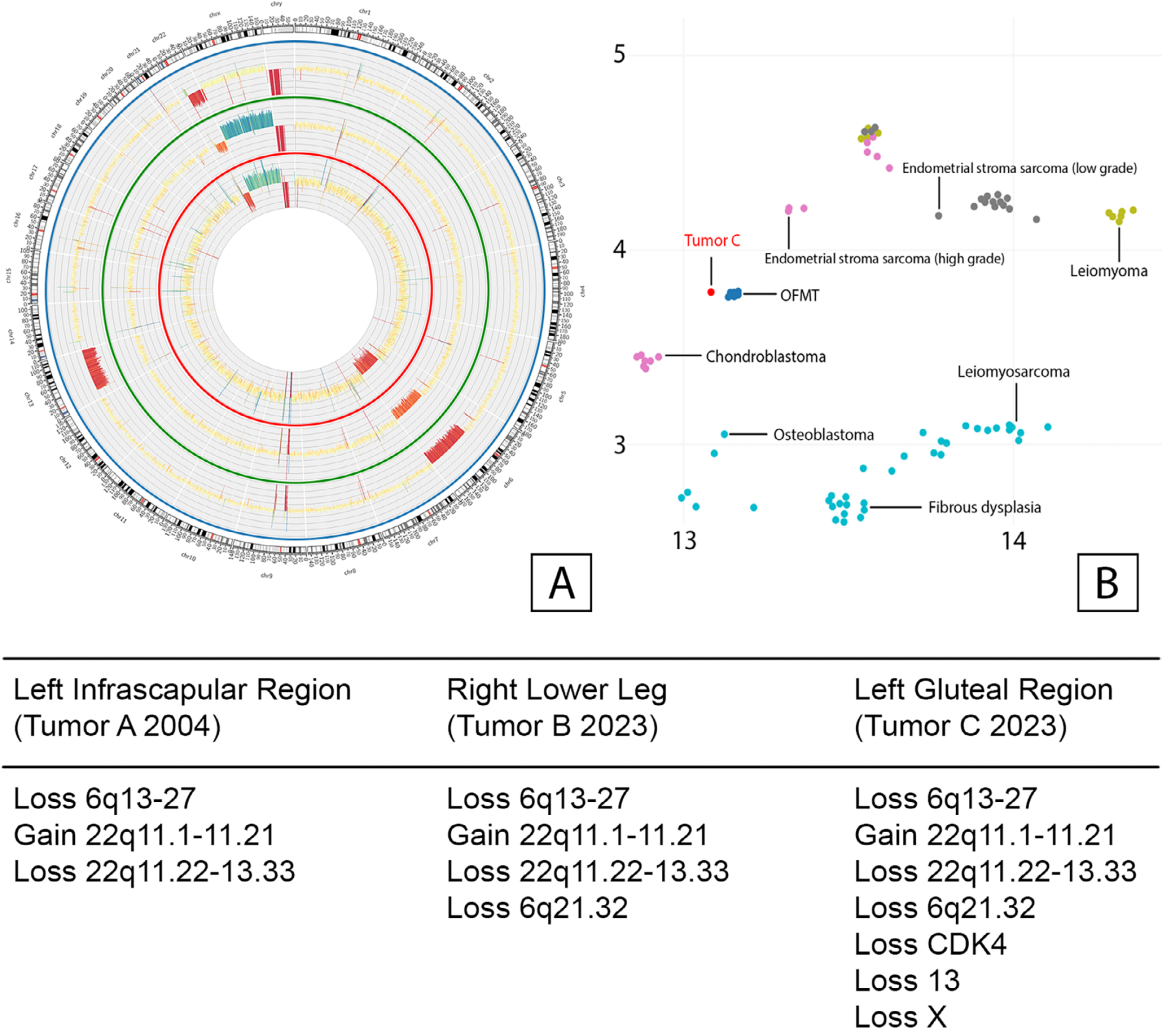
Copy number variation and DNA methylation analysis. (A) Circos plot—the inner red circle shows the mutations of tumor A (left infrascapular region, 2004). The middle green circle shows the mutations of tumor B (right lower leg, 2023) and the outermost blue circle the mutations of tumor C (left gluteal region, 2023). (B) Joint t‐distributed stochastic neighbor embedding (t‐SNE) demonstrating clustering of tumor C within the OFMT methylation class.

To evaluate the possibility of late metastatic disease versus a multifocal sequential appearance of “independent” OFMTs in February 2024, a copy number variation (CNV) analysis was carried out on all three lesions (2004, 2023/1, and 2023/2). In the three lesions studied, a common phylogenetic root was observed, along with additional CNV in the tumors removed in 2023. Therefore there is very strong evidence that the lesions 2023 are late metastases from the 2004's OFMT. A circos plot was created to illustrate the fact more clearly and to facilitate a deeper understanding of the circumstances (Figure 2A).

Furthermore, DNA methylation profiling was performed using Illumina Infinium MethylationEPIC BeadChip technology. DNA from our FFPE samples was prepared with the Illumina Infinium HD FFPE DNA Restore Kit and bisulfite conversion was performed using the EpiTect Bisulfite Kit (Qiagen). After amplification, fragmentation and hybridization of the DNA with the Illumina Infinium MethylationEPIC BeadChip, an Illumina NextSeq 550 device was used for scanning of the arrays. The results were analyzed with the Epignostix sarcoma classifier v13.1 (https://app.epignostix.com, accessed on October 3, 2025).

The classifier assigned the tumor sample (tumor C) to the OFMT methylation class with a calibrated score of 0.998, confirming a high‐confidence match. This result provides independent epigenetic confirmation that the analyzed lesion clusters within the OFMT reference group in the Heidelberg sarcoma classifier and supports the molecular and histologic diagnosis forming a coherent molecular profile consistent with OFMT [Figure 2B]. In addition, methylation data was also put through EpiDiP to generate a t‐distributed stochastic neighbor embedding (t‐SNE) visualization which established that the studied tumor clustered within the OFMT methylation class [10].

## 3 Discussion

The first description of OFMT was provided by Enzinger in 1989. OFMT primarily impacts adults, with a slightly greater occurrence found in males [4]. From a clinical standpoint, OFMT presents as a clearly defined, gradually growing tumor that arises in the muscles or subcutaneous tissues, typically causing little to no pain [11]. OFMT is now recognized as a neoplasm related to specific molecular translocations [12].

The majority, over 80% of OFMTs have recurrent gene rearrangements, predominantly involving *PHF1* on chromosome 6p21, which fuses with partners such as *EP400, MEAF6*, and *EPC1*. Some examples of new combinations of genetic material are *ZC3H7B::BCOR, CREBBP::BCOR1*, and *KDMA2::WWTR1*. Most of these genes are involved in epigenetic control and histone modification. The utilization of fluorescence in situ hybridization (FISH) to detect *PHF1* rearrangements is a valuable diagnostic tool; however, it is challenging in instances [13].

In our case we used a targeted RNA sequencing, by using the Archer solid FUSIONPlex RNA panel, to demonstrate the *EP400:PHF1* fusion. It can be concluded that the identification of these fusions serves as a reliable indicator to confirm the diagnosis of OFMT besides histology, immunohistochemistry and radiological imaging. Our CNV analysis showed gains and losses mainly affecting chromosomes 6 and 22 (Figure 2A).

All three tumors A–C demonstrated loss of 22q11.22‐13.33 and gain of 22q11.1‐11.21 on chromosome 22 as well as a loss of 6q13‐27 on chromosome 6.

Tumors B and C in addition demonstrated a loss on chromosome 6 (6q21.32), whereas in tumor C additional losses of CDK4 on chromosome 12, of chromosome 13 and the X‐chromosome were seen.

The similarities in CNV patterns especially the shared losses and gains on chromosomes 6 and 22 suggest a clonal relationship of these three tumors. The three tumors have a common phylogenetic root with additional CNV in the metastases.

The additional losses of CDK4, chromosome 13 and the X‐chromosome suggest tumor progression that could influence its biological behavior as well as the potential for further metastasis. Particular attention should be paid especially to the loss of CDK4. This protein plays a crucial role in mediating the progression from the G1 phase to the DNA synthesis phase in the cell [14].

Recently, Brown et al. have shown a similar approach in their study on the progression of breast cancer [15].

Hofvander et al. [16] analyzed several cases of OFMTs with regard to transcriptomic features, gene fusion as well as copy number status and suggested that OFMT develops through gene fusions that have extensive epigenetic consequences.

Their data suggest that all types of OFMT share a similar spectrum of gene fusions. Also, rather than being separate entities, atypical and malignant OFMTs most likely represent progression‐related variants of typical OFMTs. It could be suggested that a deletion affecting parts of chromosome 13 could serve as an indicator for atypical and malignant OFMTs. Such deletions were exclusively observed in these types of tumors in their study. Also a loss of the RB1 gene was specifically observed in atypical or malignant OFMTs. Typical OFMTs exhibited a median of two imbalances per case, in contrast to six in atypical and seven in malignant lesions. The most frequently affected chromosome band was 6p21. We had a loss of 6q21.32 in tumors B and C. They demonstrated similar results in their circos plot to our cases in SNP array analysis, showing losses in chromosomes 6, 22, and 13 [16].

In addition to the fusion and CNV analyses, the inclusion of DNA methylation profiling provides an additional molecular layer confirming the epigenetic identity of the lesion within the OFMT class.

Although genomic complexity increased in the metastatic lesions, the methylation classifier still assigned the tumor to the OFMT methylation class. This suggests that the epigenetic signature of OFMT may remain conserved during tumor evolution, even in the presence of secondary genetic alterations.

Future integrative studies combining methylome and DNA‐based profiling could identify reproducible biomarkers of aggressive behavior or late metastatic potential in otherwise typical OFMT.

## Ethics Statement

Ethical approval was obtained from the Ethics Committee of the Medical University of Graz.

## Consent

A written informed consent was obtained from the patient for publication of this report and any accompanying images.

## Conflicts of Interest

The authors declare no conflicts of interest.

## Data Availability

No new datasets were generated during the current study. All relevant clinical information is included within the article.
